# Ultrasound-shear-assisted enzymatic preparation of α-glucosidase inhibitory peptides from *Juglans sigillata* D. and elucidation of their molecular inhibitory mechanism

**DOI:** 10.1016/j.ultsonch.2026.108034

**Published:** 2026-09-02

**Authors:** Xingjing Zhang, Boxiao Wu, Baitong Zhu, Zhongxing Chu, Quankang Liu, Chang Zou, Ting Ma, Feijun Luo, Yaping Zhou, Delu Ning

**Affiliations:** a State Key Laboratory of Woody Oil Resources Utilization, Central South University of Forestry and Technology, Changsha 410004, China; b Key Laboratory of National Forestry and Grassland Administration on Walnut, Yunnan Academy of Forestry and Grassland, Kunming 650201, China; c Yunnan Technology Innovation Center of Woody Oil, Yunnan Academy of Forestry and Grassland, Kunming 650201, China

**Keywords:** *Juglans sigillata D.*, Ultrasound-shear-assisted enzymatic hydrolysis, α-Glucosidase-inhibitory peptides, α-Glucosidase inhibitory activity

## Abstract

This study aimed to efficiently valorize protein resources from *Juglans sigillata* D. by employing ultrasound-shear-assisted enzymatic hydrolysis to generate α-glucosidase-inhibitory peptides. The process parameters were optimized using response surface methodology, and the structural basis underlying this enhanced inhibitory activity was further elucidated through multiscale structural characterization and molecular docking analysis. The optimal conditions were determined as follows: ultrasound treatment time of 21  min, ultrasonic power of 400  W, shear speed of 12000 r/min, and enzyme loading of 12 KU/g. Under these conditions, the α-glucosidase inhibition rate of the hydrolysate reached 54.38 ± 0.77 %, which was significantly higher than that obtained by enzymatic hydrolysis alone, with the latter showing an inhibition rate of 45.82 ± 3.28 % (*p* < 0.05). Structural analyses indicated that the combined ultrasound–shear treatment modified the conformational structure and increased the surface hydrophobicity of walnut protein, potentially enhancing protease accessibility and facilitating the release of α-glucosidase-inhibitory peptides. In total, 4492 peptides were identified by LC-MS/MS, from which 20 candidate peptides were prioritized using a BIOPEP-aided, two-stage *in silico* workflow. Molecular docking was subsequently used to compare the predicted binding modes and docking scores of selected peptides with α-glucosidase. The peptides FFPGSP, FGPSQPF, and APSKDAPMF were synthesized and experimentally validated. Their IC_50_ values were 1745, 5393, and 4090  μM, respectively. These findings show that combining peptide identification, bioinformatics screening, molecular docking, and *in vitro* validation can help facilitate the preliminary discovery and selection of α-glucosidase-inhibitory peptides. This study provides a basis for the high-value utilization of *J. sigillata* protein and the development of natural peptides with potential α-glucosidase-inhibitory activity.

## Graphical abstract

**Figure:**
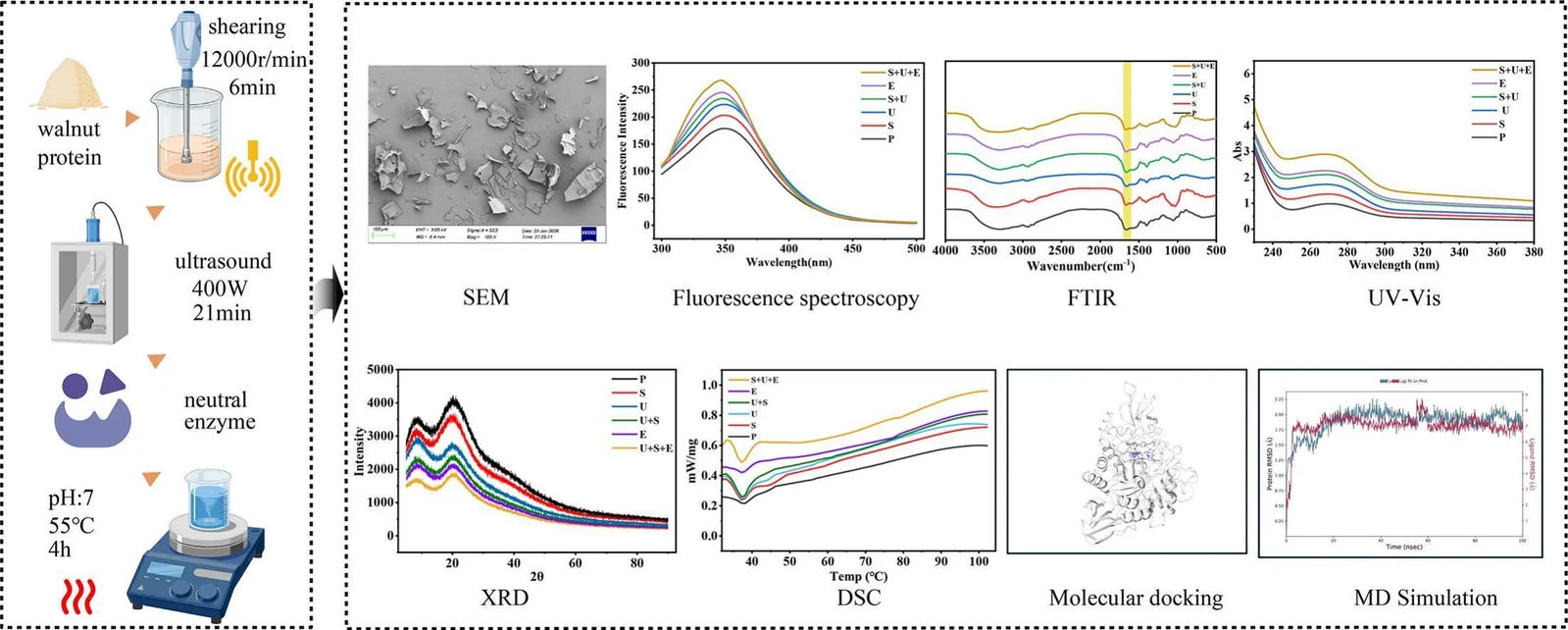

## 1 Introduction

Globally, type 2 diabetes mellitus (T2DM) has emerged as a pervasive chronic metabolic disorder. The disruption of postprandial glucose homeostasis is recognized as a critical determinant of systemic metabolic health [1], [2]. As a primary therapeutic target for regulating blood glucose, α-glucosidase catalyzes the hydrolysis of complex carbohydrates into absorbable monosaccharides; consequently, its excessive activity is directly linked to rapid postprandial glycemic spikes [3]. Pharmacological α-glucosidase inhibitors can effectively attenuate postprandial hyperglycemia; however, their long-term use is frequently associated with adverse side effects, including gastrointestinal distress and weight fluctuations, which limit their clinical utility [4], [5], [6]. Therefore, identifying safe, natural, food-derived alternatives for T2DM intervention has garnered significant research interest. Bioactive peptides may possess favorable properties, including good biocompatibility, potential for efficient absorption, and generally low toxicity, making them promising candidates for the development of functional α-glucosidase-inhibitory ingredients. *Juglans sigillata* D. (Yunnan deep-pattern walnut) is an important woody oil crop native to Southwest China. Defatted walnut meal contains approximately 46.14 % protein with a balanced amino acid profile. This composition renders it an ideal substrate for bioactive peptide production [7].

Although enzymatic hydrolysis is a standard technique for producing bioactive peptides, its efficacy is often hindered by the inherent structural constraints of the substrate [8]. Specifically, *J. sigillata* protein possesses a highly compact conformation and extensive molecular aggregation, thereby restricting protease accessibility and leading to suboptimal hydrolysis efficiency [9]. The protein is rich in intra-and inter-molecular disulfide bonds and buried hydrophobic residues, such as Leu, Pro, and Phe, while containing relatively few surface-exposed polar and charged groups. This dense, semi-crystalline matrix reduces water penetration and conformational flexibility. It also physically blocks protease diffusion into the interior. Consequently, the exposure of specific cleavage motifs is decreased, which suppresses enzymatic hydrolysis efficiency and bioactive peptide release [8], [9].

To overcome these barriers, ultrasonication (US) and high-speed shearing (HSS) have been employed as eco-friendly pretreatments. US cavitation disrupts non-covalent interactions and may promote the exposure or rearrangement of disulfide bonds, thereby unfolding proteins and exposing buried cleavage sites [[10], [11], [12]]; HSS mechanically fragments aggregates, reduces particle size, and increases surface area [[13], [14], [15]]. Their synergistic action exhibits a functional hierarchy: ultrasonication primarily disrupts intramolecular interactions and unfolds the protein conformation, while high-speed shearing mainly disintegrates supramolecular aggregates and increases the accessible surface area [[16], [17]]. This coordinated mechanism maximizes protease-substrate interactions. Importantly, process parameters critically modulate not only the extent of hydrolysis but also the peptide size distribution and sequence motifs: moderate sonication (20 min, 400 W) and appropriate enzyme dosage (12–14 KU/g) favor the release of bioactive peptides, whereas excessive treatment can over-degrade active sequences or cause protein over-denaturation [[18], [19], [20]]. An optimal shear speed (12,000 r/min) ensures sufficient particle size reduction without inducing insoluble aggregation [[13], [15]]. However, the application of coupled ultrasound-high-shear synergy to prepare α-glucosidase inhibitory peptides from *J. sigillata* protein remains unexplored. Recently, ultrasound-assisted enzymolysis has also been successfully applied to marine animal proteins, such as Perinereis aibuhitensis, significantly improving hydrolysis efficiency and peptide yield when combined with response surface methodology (RSM) and back-propagation-genetic algorithm (BP-GA) modeling [21].

Ultrasound-assisted enzymolysis has been widely reported for the modification of plant proteins, including walnut protein [[9], [17], [20]]. Specifically, Huo et al. [20] demonstrated that ultrasound-assisted enzymolysis significantly enhances the antioxidant activity of walnut protein-derived peptides, with an optimal ultrasonic power window of approximately 180 W. Zhao et al. [17] reported that sequential ultrasound and high-speed shear pretreatments effectively reduce walnut protein particle size (from >800 nm to < 500 nm) and increase surface hydrophobicity and free sulfhydryl content. A recent review has also summarized various modification strategies, including ultrasound-assisted limited enzymolysis and glycosylation, which improve walnut protein solubility [9]. Nevertheless, these studies did not systematically characterize the multiscale structural evolution of walnut protein, ranging from secondary conformation to quaternary organization, under coupled ultrasound-high-shear treatment. More importantly, the integration of bioinformatics-driven virtual screening with molecular dynamics (MD) simulations for α-glucosidase inhibitory peptide discovery from walnut protein has not been reported. In addition, the complementary effects of ultrasonication at the molecular level and high-speed shearing at the supramolecular level remain underexplored. Collectively, these studies demonstrate that US and HSS can be used to tune particle size, surface hydrophobicity, sulfhydryl/disulfide balance, and solubility across different protein sources, yet a systematic comparison of single, dual and coupled ultrasound-high-shear synergy treatments in walnut protein, and their impact on peptide composition and α-glucosidase-inhibitory activity, is still lacking.

The present work offers three distinct innovations. One is a coupled US-HSS pretreatment paradigm that maximizes synergistic effects beyond sequential or single treatments. Another is systematic multiscale structural characterization (SEM, FTIR, XRD, DSC, etc.), which reveals a transition from semi-crystalline to amorphous states. The third is a high-throughput bioinformatics screening strategy (PeptideRanker, ToxinPred, AllerTOP, molecular docking, and MD simulations) for rapid identification of α-glucosidase inhibitory peptides. Based on these innovations, this study employed US-HSS assisted enzymolysis to prepare α-glucosidase inhibitory peptides from *J. sigillata* protein. Using α-glucosidase inhibition as the primary indicator, the coupled pretreatment and hydrolysis conditions were systematically optimized, and the associated multiscale structural changes and structure–activity relationships were elucidated.

## 2 Materials and methods

### 2.1 Materials and reagents

Walnut (*Juglans sigillata* D.) protein powder was sourced from Yunnan Province, China, and supplied as a defatted powder. The defatted protein powder was vacuum-sealed and stored at 4 °C under dry conditions. Trypsin, alkaline protease (AP), neutral protease (NP), and papain were purchased from Nanning Donghenghuadao Biotechnology Co., Ltd. (Guangxi, China). α-Glucosidase was obtained from Beyotime Biotechnology (Shanghai, China). All other chemical reagents utilized in this study were of analytical grade.

### 2.2 Preparation of *J. sigillata* protein hydrolysates

To minimize lipid interference during proteolysis and subsequent peptide purification, the *J. sigillata* protein powder was subjected to a pre-defatting process. The resulting defatted powder was dispersed in distilled water to achieve a 4% (w/v) suspension. Prior to pH adjustment and enzyme addition, the protein suspension was subjected to sequential ultrasonic and high-speed shear pretreatments. Ultrasonication was performed using an ultrasonic processor (JY92-IIN, Ningbo Scientz Biotechnology Co., China) equipped with a titanium probe (6 mm diameter, operating frequency 20 kHz). The ultrasonic power was set at 400 W and applied in pulse mode (2.0 s on, 0.6 s off) in an ice-water bath to minimize thermal effects. High-speed shearing was conducted using a digital homogenizer (T25, IKA, Germany) with an 18 mm diameter rotor-stator generator at 12,000 r/min for 6 min. After pretreatment, the pH and temperature were adjusted to optimal conditions for each enzyme using 0.1 mol/L HCl or NaOH. Subsequently, the proteases (trypsin, papain, AP, or NP) were added at a standardized enzyme-to-substrate ratio of 14 KU/g. Following 4 h of enzymatic hydrolysis, the reaction was terminated by heating in a 100 °C water bath for 10 min to ensure complete enzyme inactivation. The hydrolysate was then cooled to 25 °C and centrifuged at 8,000 × g (4 °C, 25 min). The resulting supernatant was collected, dispensed into amber polypropylene tubes in 5–10 mL aliquots, and immediately frozen at -20 °C in the dark. All hydrolysates were used within 2 weeks of preparation, and each aliquot was thawed only once to avoid repeated freeze-thaw cycles before further analyses [22].

### 2.3 Degree of hydrolysis (DH) measurement

The DH of *J. sigillata* protein was evaluated using the o-phthalaldehyde (OPA) spectrophotometric assay, following the protocol of Cheng et al. [23] with minor modifications. Briefly, 400 μL of the sample was reacted with 3 mL of OPA reagent for 2 min at room temperature in the dark. The absorbance was recorded at 340 nm. The concentration of free amino groups was determined to calculate the DH, with all experiments conducted in triplicate.

### 2.4 α-Glucosidase inhibitory activity assay

The α-glucosidase inhibitory potential of the *J. sigillata* peptides was determined using a commercial assay kit according to the manufacturer’s instructions. The reaction mixture, containing the peptide sample, enzyme solution, and specific substrate, was incubated at 37 °C. The absorbance was measured using a UV-visible spectrophotometer, and the inhibition rate was calculated based on the provided formula.

### 2.5 Single-factor experiments

*J. sigillata* protein was suspended in ultrapure water and hydrolyzed at pH 7.0 and 55 °C for 4 h. With the α-glucosidase inhibition rate as the evaluation index, single-factor experiments were conducted to optimize the process parameters. All preliminary treatments were performed using the same ultrasonic processor (20 kHz, 6 mm probe) and shear homogenizer with an 18 mm rotor-stator generator as described in Section 2.2, and ultrasonication was conducted in an ice-water bath with the same pulse mode to control temperature. The shear speed was fixed at 12000 r/min and shear treatment time at 6 min. These shear conditions were chosen based on preliminary trials and previous reports on walnut and legume proteins [[14], [17]], which showed that 10000–12000 r/min for 5–10 min is sufficient to disrupt aggregates and reduce particle size without causing excessive foaming or protein denaturation. Specifically, the effect of ultrasound treatment time from 10 to 30 min was investigated with an enzyme dosage of 14 KU/g and ultrasonic power of 200 W. Subsequently, while maintaining ultrasonic power at 200 W and time at 20 min, the influence of enzyme dosage from 10 to 18 KU/g was explored. Finally, with the ultrasound treatment time fixed at 20 min and the enzyme dosage fixed at 14 KU/g, the effect of ultrasonic power was investigated over the range of 300–500 W.

### 2.6 Response surface methodology (RSM) design

Based on the results of the single-factor experiments, three independent variables including ultrasound treatment time, ultrasonic power, and enzyme dosage were further optimized using a Box-Behnken design via Design-Expert 13.0 software. The α-glucosidase inhibitory activity was utilized as the response value to determine the optimal conditions for preparing *J. sigillata* α-glucosidase inhibitory peptides.

### 2.7 Particle size and zeta potential measurements

Dynamic light scattering (DLS) was employed to determine the particle size distribution and zeta potential of the samples at 25 °C using a Zetasizer Nano ZS90 (Malvern Instruments, UK). Samples were diluted to 0.1% (w/v) with ultrapure water to ensure uniform dispersion. The refractive index of water was set at 1.330, and each sample was analyzed in triplicate.

### 2.8 Scanning electron microscopy (SEM)

The surface microstructure of *J. sigillata* protein was observed using a field-emission scanning electron microscope (Sigma 360, ZEISS, Germany). Lyophilized protein powder was mounted onto a sample stage and coated with a thin layer of gold via sputtering. Morphological analysis was then performed following the methodology described by Zhao et al. [17].

### 2.9 Fourier transform infrared (FTIR) spectroscopy

FTIR spectra were recorded following the protocol of Zhou et al. [24]. Properly weighed samples were mixed with potassium bromide in a mass ratio of 1:100 and pressed into translucent pellets. The scanning was performed using an IRTracer-100 FTIR spectrometer (SHIMADZU, Japan) with a resolution of 4 cm^−1^ over 32 scans. The spectra were recorded in the wavenumber range from 4000–400 cm^−1^.

### 2.10 UV-visible and fluorescence spectroscopy

UV-visible absorption spectra were recorded in the range of 200–400 nm using an Agilent Cary 60 spectrophotometer, with ultrapure water serving as the blank reference. For intrinsic fluorescence measurements, samples were diluted to a concentration of 1 mg/mL. The spectra were captured using an F-4500 fluorescence spectrophotometer (Hitachi, Japan) with an excitation wavelength of 280 nm and an emission spectrum collected from 300 to 500 nm. Both excitation and emission slit widths were maintained at 5 nm, with a scanning speed of 100 nm/min.

### 2.11 Surface hydrophobicity (*H*_0_)

The *H*_0_ of the samples was determined using the ANS-binding method as described by Hu et al. [25]. Samples were diluted in 0.01 mol/L phosphate buffer at pH 7.0, followed by centrifugation at 10,000 × g and 4 °C for 15 min. The resulting supernatants were further adjusted to a series of protein concentrations ranging from 0.0005 to 0.1 mg/mL. After the addition of the ANS fluorescent probe, the fluorescence intensity was measured at an excitation wavelength of 390 nm and an emission range of 400–600 nm. The value of *H*_0_ was defined as the initial slope of the linear regression curve of relative fluorescence intensity against protein concentration.

### 2.12 Free sulfhydryl (SH) content

The free SH content was determined using Ellman’s reagent according to a previously reported method [26]. All samples were standardized to a uniform concentration and mixed with an equal volume of Tris-glycine buffer. Immediately after the addition of Ellman’s reagent, the mixture was incubated in the dark at room temperature for 1 h. The absorbance was then measured at 412 nm using a UV-visible spectrophotometer.

### 2.13 X-ray diffraction (XRD)

XRD patterns were acquired using an X'Pert3 Powder X-ray diffractometer (Malvern Panalytical, UK). The scanning was performed at a rate of 5°/min over a 2*θ* range from 5° to 90°.

### 2.14 Differential scanning calorimetry (DSC)

Thermal stability was evaluated using a DSC 214 Polyma (NETZSCH, Germany). Approximately 10 mg of lyophilized powder was sealed in an aluminum crucible and heated under a continuous nitrogen atmosphere.

### 2.15 Identification and bioinformatics analysis of *J. sigillata* peptides

Protein extracts from *J. sigillata* were sequentially processed by dissolution, ultrafiltration, reductive alkylation, desalting and lyophilization prior to peptide profiling. Peptide sequencing was performed using LC-MS/MS coupled to a C18 reversed-phase column with gradient elution, and raw mass spectrometry data were processed in MaxQuant (v.2.4.14.0) against a species-specific protein sequence database.

All 4492 identified peptide sequences were subjected to tiered *in silico* screening targeting α-glucosidase inhibitory activity, drawing on previously reported screening strategies [[27], [28]]. PeptideRanker (http://distilldeep.ucd.ie/PeptideRanker/) was first used to generate quantitative scores, and a cutoff value of 0.80 was applied to prioritize high-potential candidates. In parallel, physicochemical and safety profiling was performed using multiple independent web servers, including PepCalc (http://pepcalc.com/) for hydrophobicity calculation, ToxinPred (https://webs.iiitd.edu.in/raghava/toxinpred/index.html) for acute toxicity prediction, AllerTOP (http://www.ddg-pharmfac.net/AllerTOP) for preliminary allergenicity assessment, and AnOxPePred (http://services.bioinformatics.dtu.dk/service.php) for radical-scavenging antioxidant prediction.

To construct rigorous database-backed filtering criteria, all pre-screened candidate peptides underwent secondary database-based verification on the BIOPEP-UWM bioactive peptide database (BIOPEP-UWM) platform via two sequential analytical pipelines. The Profiles of Potential Biological Activity module was first used to identify potential α-glucosidase-inhibitory motifs. These motifs included the core dipeptide PP and continuous stretches of at least three amino acid residues homologous to experimentally validated inhibitory peptides in the BIOPEP-UWM database; DPP-IV inhibitory sequences retrieved from the BIOPEP Virtual Peptides library were only cited to interpret multi-pathway hypoglycemic potential and were not set as screening exclusion standards. Following motif characterization, the embedded FIND module of BIOPEP-UWM was applied to align candidate sequences against the built-in allergenic epitope dataset, delivering orthogonal verification consistent with the preliminary allergenicity outputs from AllerTOP (http://www.ddg-pharmfac.net/AllerTOP). Only peptides satisfying both tiers of screening were retained for subsequent molecular docking simulations. First, candidates must meet all primary multi-index requirements, namely high predicted bioactivity, appropriate hydrophobicity profiles, and no computationally predicted toxic or allergenic signals from the standalone web tools. Second, all candidates should pass secondary BIOPEP-UWM validation with detectable conserved α-glucosidase inhibitory motifs and no matched allergenic epitopes [29].

### 2.16 Determination of half-maximal inhibitory concentration (IC_50_) of synthetic peptides

The three top-ranked peptides (FFPGSP, FGPSQPF, and APSKDAPMF; purity > 95%) were commercially synthesized by Shanghai Gil Biochemical Co., Ltd. (Shanghai, China). The lyophilized peptides were stored at −20 °C in the dark and dissolved in an appropriate solvent to prepare stock solutions. The stock solutions were then diluted with the assay buffer (α-GC Assay Buffer, provided in the kit) to yield a range of final concentrations: 500, 1500, 2500, 3500, 4500, 5500, 6500, 7500, and 8500 μM. The concentration range was selected based on preliminary trials to cover the full dose-response spectrum from minimal to maximal inhibition. The α-glucosidase inhibitory activity at each concentration was measured following the same protocol as described in Section 2.4. The percentage inhibition was plotted against the logarithm of peptide concentration (Log_10_ Concentration), and the IC_50_ values were calculated by non-linear regression fitting using a variable-slope (four-parameter logistic) model in GraphPad Prism 9.0.

### 2.17 Molecular docking

To probe the binding geometry between *J. sigillata* peptides and α-glucosidase, molecular docking was implemented using the Schrödinger Suite (Schrödinger, LLC). The crystalline coordinates of the receptor were obtained from the RCSB Protein Data Bank (PDB ID: 3WY1; https://www.rcsb.org/structure/3WY1). Structural preparation of the enzyme was handled via the Protein Preparation Wizard, involving the optimization of bond orders, incorporation of hydrogen atoms, and energy minimization within the OPLS3 force field. The docking grid was centered on the catalytic site of the receptor. Three-dimensional structures of the peptide ligands were prepared using LigPrep, including the generation of appropriate protonation states and energetically reasonable conformations. Docking calculations were performed using Glide in Standard Precision (SP) mode, with structural visualizations produced using PyMOL (v.2.0) (https://pymol.org/2/).

### 2.18 Molecular dynamics (MD) simulation

Computational MD simulations were conducted with the Desmond engine (v.53011, Schrödinger, LLC). Initially, the binding configurations of the complexes were refined using the Protein Preparation Wizard to optimize the hydrogen-bonding network and eliminate unfavorable steric contacts. The systems were immersed in an orthorhombic buffer box utilizing the TIP3P water model, with atomic interactions parameterized by the OPLS3 force field. To maintain electrical neutrality and mimic the physiological milieu, Na^+^ ions were introduced. The simulation proceeded under default NPT conditions to capture the dynamic evolution of the system. Post-simulation analysis was performed using the Simulation Interactions Diagram, and the Root Mean Square Deviation (RMSD) was monitored to quantify the temporal stability and structural variations of the complexes.

### 2.19 Statistical analysis

All experiments were performed at least in triplicate using independently prepared biological samples. Results are expressed as mean ± standard deviation (SD). One-way analysis of variance (ANOVA) was conducted for all treatments, followed by Tukey’s multiple comparison test to determine significant differences (*p* < 0.05). Statistical analyses were carried out using GraphPad Prism 9.0 (GraphPad Software, San Diego, CA, USA).

## 3 Results and discussion

### 3.1 Screening of proteases

Proteolysis using various enzymes is the standard approach for liberating *J. sigillata*-derived peptides. Common biocatalysts in this field include neutral protease, alkaline protease, trypsin, and papain. To evaluate the extent of protein hydrolysis and the efficacy of these enzymatic treatments, the degree of hydrolysis (DH) was monitored as a primary functional indicator. Generally, a higher DH may be associated with a lower average molecular weight of the resulting peptides; however, the biological activity also depends on peptide sequence, composition, and structure. [30]. In the current investigation, we comparatively assessed the hydrolytic performance of four proteases: neutral, alkaline, trypsin, and papain. Analysis of the hydrolysis profiles (Fig. 1A) and the corresponding α-glucosidase inhibition (Fig. 1B) reveals that neutral protease treatment resulted in the highest degree of hydrolysis and the strongest α-glucosidase-inhibitory activity. Specifically, the DH achieved with neutral protease was 20.80% and 35.82% higher than those obtained with alkaline protease and papain, respectively. Furthermore, its resultant hydrolysate attained an α-glucosidase inhibition level of 57.07 ± 1.37%. This robust performance is likely governed by the endopeptidase activity of neutral protease, which targets internal peptide bonds to facilitate thorough degradation [31]. Therefore, neutral protease was selected for all subsequent optimization phases.

**Fig. 1:**
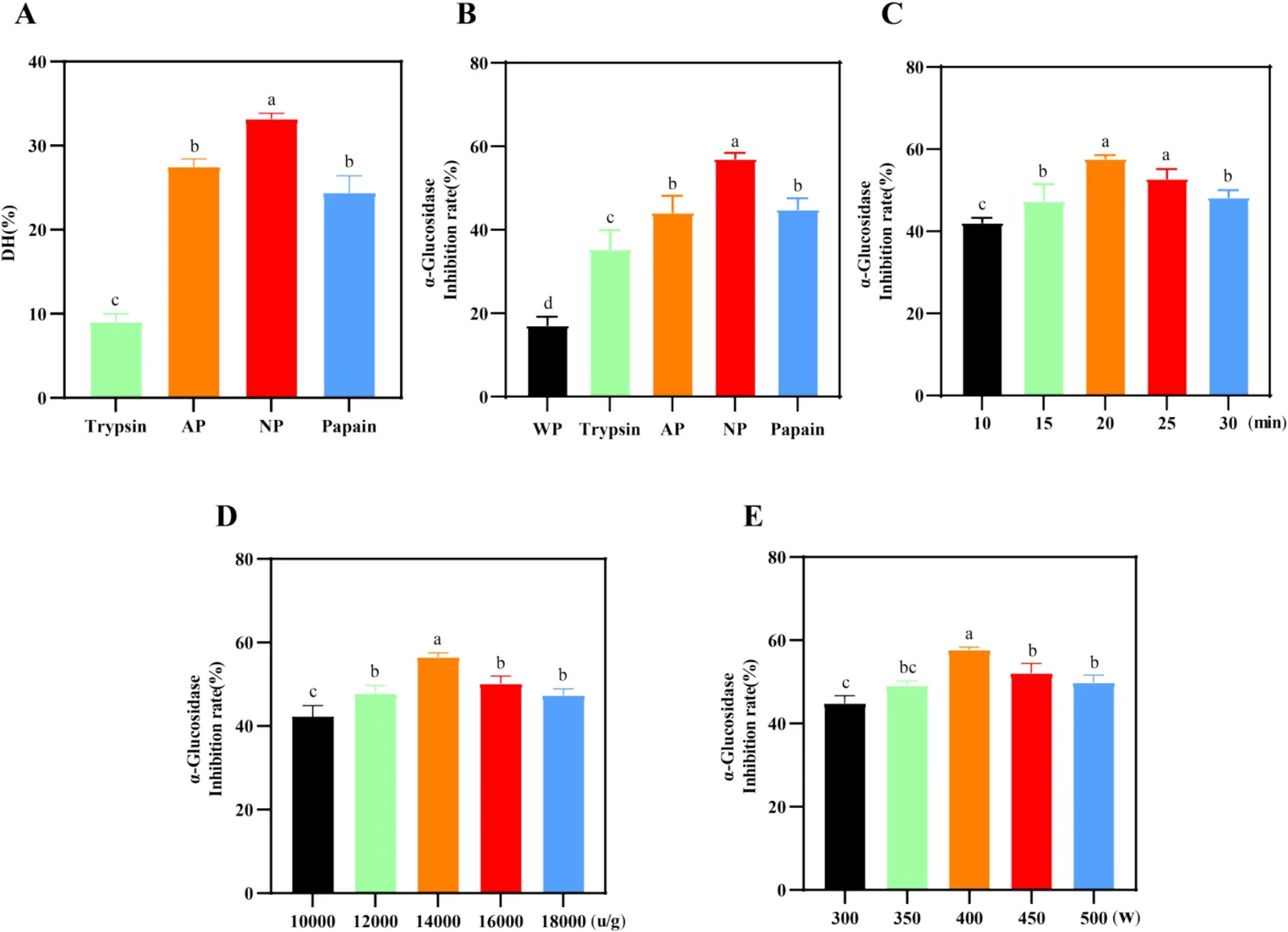
Screening of proteases and optimization of single-factor enzymatic conditions. (A) Degree of hydrolysis (DH) of *J. sigillata* protein (P) as affected by different proteases. (B) α-Glucosidase inhibitory activity of P and its hydrolysates (WPHs) prepared with different enzymes. (C-E) Effects of (C) ultrasound treatment time, (D) enzyme dosage, and (E) ultrasonic power on the α-glucosidase inhibitory activity of WPHs. Data are expressed as mean ± SD (n = 3). Different lowercase letters (a-d) indicate significant differences (*p* < 0.05) according to one-way ANOVA followed by Tukey’s test. (Here and in all subsequent figures, the following abbreviations are used: P, untreated protein; S, high-speed shearing alone; U, ultrasonication alone; S + U, combined ultrasonication and high-speed shearing; E, enzymatic hydrolysis alone; S + U + E, ultrasound-shear-assisted enzymatic hydrolysis).

### 3.2 Analysis of single-factor experimental results

To investigate the influence of ultrasound treatment time, experiments were conducted using 14 KU/g enzyme, a shear speed of 12,000 r/min, a shearing time of 6 min, and an ultrasonic power of 200 W. These shear conditions were selected based on preliminary screening. As illustrated in Fig. 1C, α-glucosidase inhibition showed a 1.37-fold increase during the first 20 min of ultrasound exposure compared to the untreated control. However, extending the duration beyond this point led to a significant decrease in activity, especially evident between the 20-min and 30-min time points (*p* < 0.01). This decline is attributed to excessive ultrasound-induced unfolding and subsequent re-aggregation of the protein matrix, which reduces protease accessibility, as well as further fragmentation of already-released inhibitory peptides into shorter fragments with reduced activity [[11], [18], [19], [20]].Because the protease was introduced after ultrasonication and the entire sonication process was conducted in an ice-water bath, the enzyme was not exposed to ultrasonic fields, and substantial thermal inactivation was unlikely.

The impact of enzyme loading was subsequently examined following a 6-min shear (12000 r/min) and 20-min sonication (200 W). Data in Fig. 1D indicated that an inhibition rate of 56.51 ± 1.02% was achieved at 14 KU/g. While moderate enzyme concentrations effectively release bioactive fragments, exceeding this threshold leads to a decline in inhibitory activity. This aligns with research by Aquino et al., who noted that exhaustive hydrolysis can eliminate DPP-IV inhibitory properties [18]. Per the mechanism suggested by Le Maux et al., the enzyme-to-substrate ratio dictates bond cleavage specificity; excessive protease may shift the kinetic balance toward the degradation of active peptides into inactive amino acids or short chains [19]. Hence, the dosage was restricted to 14 KU/g for optimal results.

Regarding ultrasonic power (Fig. 1E), the inhibitory efficacy improved progressively as the power rose to 400 W, reaching a peak of 57.67 ± 0.68%. Low-frequency sonication induces cavitation and mechanical shear that restructure the protein matrix, reducing α-helix content and promoting molecular unfolding [11]. This structural transition facilitates greater enzyme accessibility to the protein core and enhances peptide interactions with α-glucosidase. Nonetheless, excessive power may generate intense localized heat, potentially deactivating the protease itself. This observation mirrors the findings of Huo et al., where extreme power levels diminished the antioxidant capacity of *J. sigillata* protein. Therefore, 400 W was identified as the optimal power setting [20]. Under the fixed shear conditions (12000 r/min, 6 min), the Z-average of *J. sigillata* protein was reduced from 807.1 ± 15.1 to 660.3 ± 55.3 nm without visible phase separation or excessive temperature rise, and these parameters were thus used as the mechanical baseline in all single-factor tests.

### 3.3 Statistical optimization via RSM

Building on the preliminary single-factor findings, a multivariate RSM design was implemented using Design-Expert 13 (Table S3). The following second-order polynomial model was established to correlate the α-glucosidase inhibition rate (%) with enzyme dosage (*A*), ultrasonic power (*B*), and ultrasound treatment time (*C*): Inhibition rate (%) = 57.89 + 1.07*A* + 0.3375*B* + 0.7788*C* + 0.0450*AB* + 0.4375*AC* - 0.9450*BC* - 6.32*A*^2–4^.78*B*^2^ - 4.96*C*^2^. The model statistics (R^2^ = 0.9991; adjusted R^2^ = 0.9974) indicate that the regression accounts for over 99.7% of the observed variance. The model was highly significant (*p* < 0.01). The “Adequate Precision” value of 65.8716 confirms a robust signal for navigating the experimental design space.

As visualized in Fig. 2, the interaction among enzyme dosage, ultrasonic power, and ultrasound treatment time significantly affected the α-glucosidase inhibition rate. The model-derived optimal conditions were determined as: 20.55 min duration, 402.76 W power, and 12.06 KU/g enzyme, yielding a predicted maximum of 55.90%. For practical implementation, these were adjusted to 21 min, 400 W, and 12 KU/g. Experimental validation under these refined settings yielded an inhibition rate of 54.38 ± 0.77%, which was reasonably close to the predicted value of 55.90%, supporting the predictive performance of the RSM model. These results support the use of the RSM-optimized process as an effective strategy for preparing α-glucosidase-inhibitory *J. sigillata* peptides. Although the single-factor experiments identified 14 KU/g as the enzyme dosage yielding the highest inhibition rate under fixed sonication conditions, the RSM model predicted a slightly lower optimum of 12 KU/g when enzyme dosage, ultrasonic power, and time were optimized simultaneously. This difference reflects the interaction among the three factors, whereby a moderate enzyme loading (12 KU/g) can be compensated by more favorable sonication conditions to achieve comparable or even higher α-glucosidase inhibition. This discrepancy also illustrates that the optimum predicted by RSM within the experimental design space can differ from the local optimum identified under fixed single-factor conditions, where factor interactions are not taken into account.

**Fig. 2:**
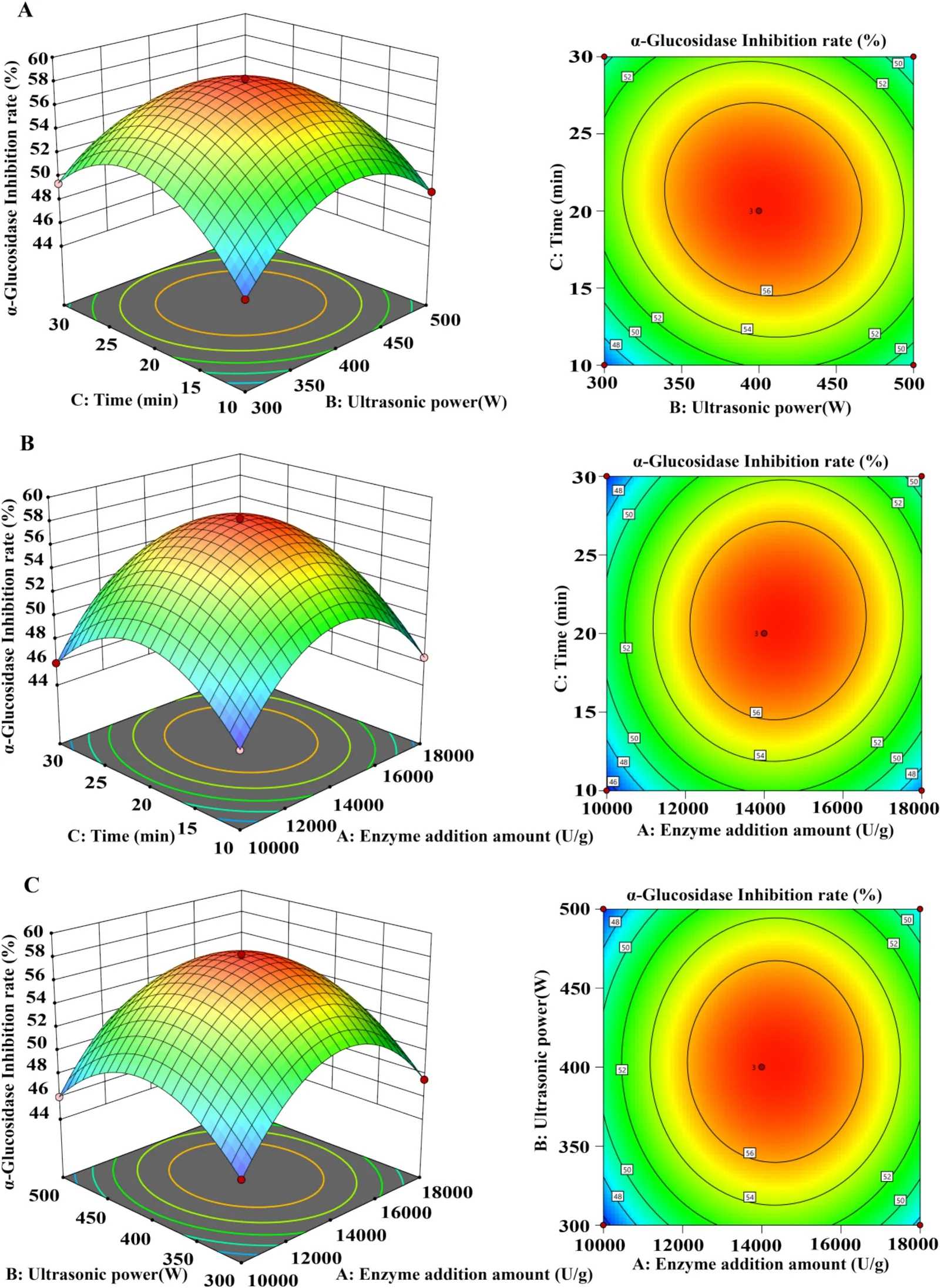
Response surface methodology (RSM) analysis of WPH preparation. 3D response surface plots and corresponding 2D contour plots illustrating the interactive effects of (A) ultrasonic power and ultrasound treatment time, (B) enzyme dosage and ultrasound treatment time, and (C) enzyme dosage and ultrasonic power on the α-glucosidase inhibitory activity. Data points for RSM fitting are mean values from triplicate experiments.

### 3.4 Cooperative effects on particle size and zeta potential

The morphological dimensions of *J. sigillata* protein particles were significantly modulated by the treatments (Fig. 3A). The control sample exhibited a Z-average particle size of 807.1 ± 15.11 nm, suggesting a state of extensive macromolecular clustering. Upon ultrasound treatment or high-speed shearing alone, these dimensions contracted to 660.3 ± 55.27 nm and 592.5 ± 20.74 nm, respectively. The combined application of these physical forces further reduced the size to 481.7 ± 34.41 nm, while the integrated ultrasound-shear-assisted enzymatic hydrolysis (S + U + E) yielded the smallest diameter of 280.6 ± 9.58 nm. These results indicate that the combined treatments were more effective than individual treatments in reducing particle size, with the highest efficacy achieved when ultrasound, high-speed shearing, and enzymatic hydrolysis were applied in sequence.

**Fig. 3:**
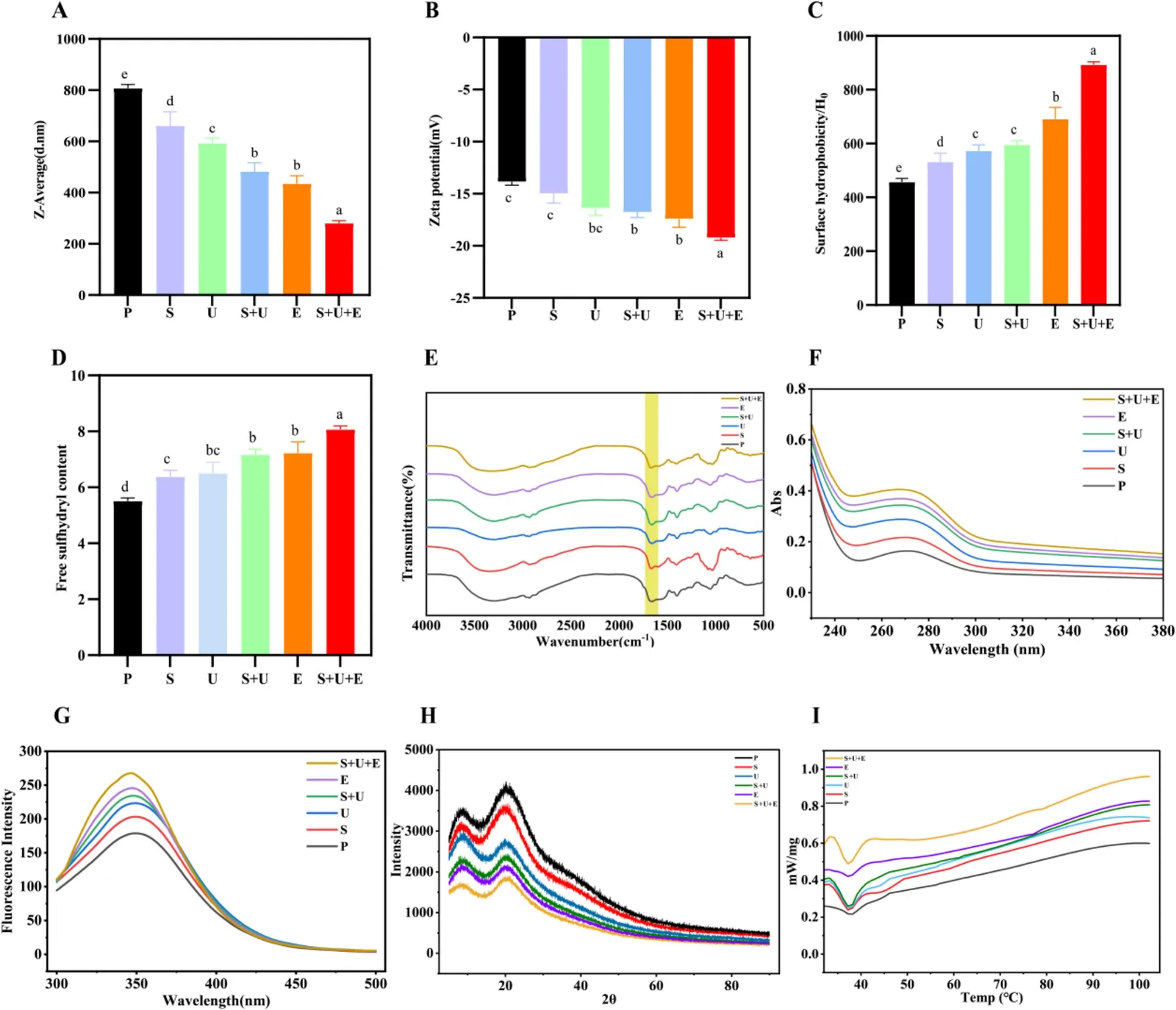
Physicochemical and structural characterization of *J. sigillata* protein under different treatments. Effects of various treatments on (A) particle size (Z-average), (B) zeta potential, (C) surface hydrophobicity (*H*_0_), and (D) free sulfhydryl content. (E-I) Structural analysis: (E) FTIR spectra (highlighting the amide I and II bands), (F) UV-vis absorption spectra, (G) intrinsic fluorescence spectra, (H) X-ray diffraction (XRD) patterns, and (I) DSC thermograms illustrating thermal stability changes. Data in panels (A-D) are expressed as mean ± SD (n = 3); Significant differences between groups are indicated by different lowercase letters (a-e) above or below the bars (*p* < 0.05).

Zeta potential serves as a key indicator of surface charge density and the electrostatic stability of the suspension [[32], [33]]. As shown in Fig. 3B, the native protein's potential was -13.84 ± 0.33 mV. Individual sonication and hydrolysis shifted this value to -16.38 ± 0.72 mV and -17.41 ± 0.82 mV, respectively. Notably, the S + U + E treatment intensified the surface negative charge to -19.23 ± 0.23 mV. This indicates an increase in the net negative surface charge and suggests enhanced electrostatic repulsion among the treated particles.

### 3.5 SEM analysis of microstructural evolution

SEM imaging (Fig. 4) captured the topographical transformations induced by the treatments. At 100 µm resolution, the untreated *J. sigillata* protein appeared as bulky, dense aggregates with irregular surface morphologies. While the individual treatments partially disrupted this compact morphology, sonication introduced distinct surface pitting and pores, leading to a more sponge-like texture. The combination of ultrasound and shearing transformed the matrix into a highly fragmented, porous state. Following subsequent proteolysis, these particles were further refined into fine, highly dispersed units. These observations show that the treatments progressively disrupted the compact protein aggregates.

**Fig. 4:**
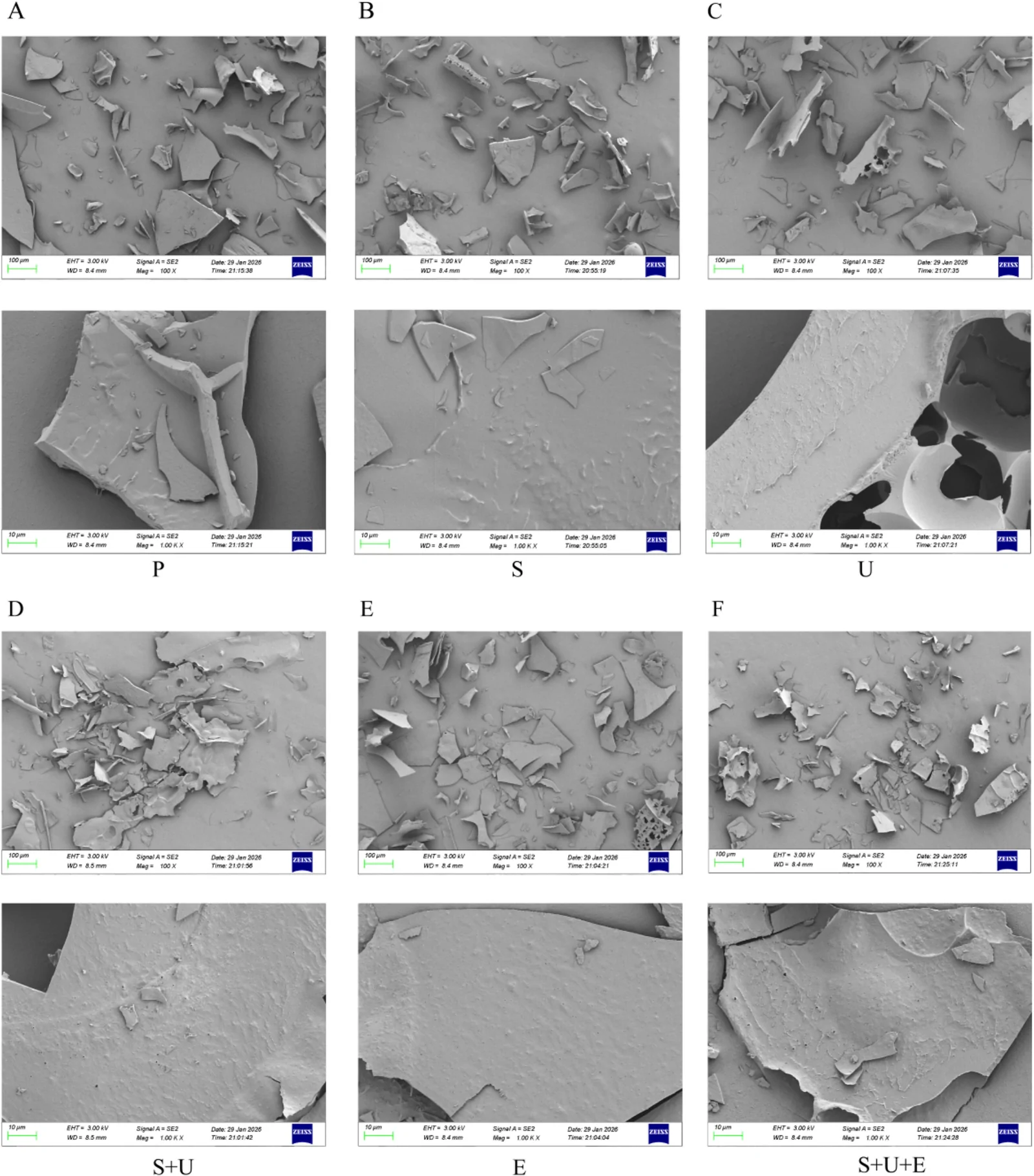
Scanning electron microscopy (SEM) micrographs of *J. sigillata* protein and its derivatives. (A) Native *J. sigillata* protein; (B) shearing; (C) ultrasound; (D) ultrasound-shearing; (E) enzymatic hydrolysis; (F) ultrasound-shearing combined with enzymatic hydrolysis. Micrographs are presented at 100× (top row) and 1000× (bottom row) magnifications.

### 3.6 Surface hydrophobicity and free sulfhydryl groups

Surface hydrophobicity (*H*_0_) reflects the exposure of hydrophobic regions during protein unfolding. As depicted in Fig. 3C, the *H*_0_ value of the S + U + E group reached 892.7 ± 11.37, significantly higher than other configurations.

The concentration of free sulfhydryl (SH) groups further elucidates the degree of structural extension (Fig. 3D). Compared to the native state (5.52 ± 0.10 µmol/g), SH content increased across all treatment groups. Standalone shearing and sonication raised the SH levels to 6.38 ± 0.23 and 6.50 ± 0.39 µmol/g, respectively, suggesting that physical stresses disrupt internal stabilizing interactions to expose buried thiols. Similar findings have been reported for corn glutenin, where ultrasonic treatment was associated with increased free sulfhydryl content and possible disruption of disulfide-related structures [12]. The S + U + E process reached a maximum SH content of 8.07 ± 0.13 µmol/g, indicating that the combined treatment promoted the exposure of previously buried sulfhydryl groups.

### 3.7 FTIR spectroscopy of secondary structures

Secondary structural shifts were monitored via FTIR, focusing on the amide I region (1600–1700 cm^−1^). As shown in Fig. 3E, the native protein displayed a characteristic absorption profile that weakened and broadened following modification. These spectral changes suggest alterations in the secondary structure of the protein.

### 3.8 Analysis of protein tertiary architecture (UV-Vis and fluorescence)

The microenvironmental changes of aromatic amino acid residues were monitored using UV–Vis absorption and intrinsic fluorescence spectroscopy. As shown in Fig. 3F, the S + U + E treatment caused a pronounced hyperchromic effect at approximately 270 nm compared with the native protein (P), suggesting increased exposure and altered local environments of aromatic residues.

The intrinsic fluorescence data further suggested changes in the microenvironment and exposure of aromatic amino acid residues, which may reflect alterations in the protein’s tertiary structure (Fig. 3G). Given that Trp, Tyr, and Phe act as sensitive endogenous fluorophores, their emission spectra are highly responsive to the polarity of their immediate surroundings [34]. The fluorescence intensity of *J. sigillata* protein followed a significant upward trend across all treatments, reaching its maximum in the S + U + E group. This enhancement indicates changes in the tertiary structure of the protein.

### 3.9 X-ray diffraction (XRD) analysis

XRD patterns (Fig. 3H) were recorded to assess the degree of structural order. All samples displayed two characteristic broad diffraction halos at 2*θ* ≈ 9° and 2*θ* ≈ 20°. Compared with the native protein (P), all modified groups showed reduced diffraction intensity and broadened peak profiles, indicating loss of ordered molecular arrangements. Notably, the S + U + E group demonstrated the lowest peak intensity and the most diffuse pattern. This suggests that the combined treatment reduced the degree of structural order.

### 3.10 Thermal properties (DSC)

The thermal transitions of *J. sigillata* protein were monitored by DSC (Fig. 3I). The native protein (P) and the enzymatically hydrolyzed group (E) exhibited shallow and broad endothermic peaks within the 20–40 °C range, characteristic of highly heterogeneous aggregates that lack organized packing. In contrast, the S + U + E group displayed a markedly higher heat-flow signal and a more concentrated endothermic peak centered near 38 °C, indicating enhanced thermal cooperativity and a more homogeneous conformational state. The elevated heat-flow curve of the S + U + E group further suggests extensive structural loosening.

### 3.11 Integrative correlation mapping and structure–activity relationship

The Pearson correlation analysis in Fig. 8 provides important insights into the structural basis of the α-glucosidase inhibitory activity of the treated *J. sigillata* protein. Particle size (Z-average) showed a strong positive correlation with zeta potential (r = 0.99, *p* < 0.05), but significant negative correlations with surface hydrophobicity (*H*_0_), free sulfhydryl (-SH) content, and DSC-derived parameters (r ≤ −0.88, *p* < 0.05). Together with the structural characterization results, these correlation patterns suggest that the ultrasound–high-shear synergistic treatment may be associated with the disruption of protein aggregates, weakening of intermolecular interactions, and conformational unfolding [17]. Accordingly, the increased exposure of buried hydrophobic residues and sulfhydryl groups may reflect a transition from a compact aggregate state to a less compact and more structurally accessible conformation.

Notably, α-glucosidase inhibitory activity was significantly negatively correlated with particle size (r = −0.92, *p* < 0.05), while showing significant positive correlations with *H*_0_ (r = 0.96, *p* < 0.05) and -SH content (r = 0.90, *p* < 0.05). This suggests that smaller protein particles, which may reflect a more dispersed and less aggregated state, may be more accessible to proteolytic enzymes, which may be associated with improved protease accessibility and a greater likelihood of generating bioactive peptides. The increase in surface hydrophobicity may enhance protein-enzyme interactions by exposing hydrophobic residues that are otherwise buried in the native structure, thereby facilitating protease access to cleavage sites [35]. Meanwhile, the elevated free sulfhydryl content may result from the partial disruption of disulfide bonds, the exposure of previously buried sulfhydryl groups, or a combination of both effects. Such structural rearrangements may be associated with a less compact molecular conformation, potentially improving substrate accessibility during hydrolysis. Together, these modifications may favor the release of low-molecular-weight peptides with greater potential for α-glucosidase inhibition.

These interpretations are consistent with the SEM and XRD results. SEM images showed that the original compact and smooth aggregates were transformed into fractured, porous, and irregular particles after the synergistic treatment, indicating obvious microstructural disintegration and a reduction in aggregation density. Such a loosened morphology may facilitate enzyme access to the protein matrix and potentially favor hydrolysis efficiency [17]. In addition, XRD analysis revealed reduced peak intensity and decreased structural order, suggesting that the ordered molecular arrangement of the protein matrix was disrupted. This decrease in apparent structural order is consistent with conformational rearrangement and the possible exposure of internal functional groups, in agreement with the observed increases in *H*_0_ and -SH content.

Mechanistically, ultrasound cavitation may disrupt non-covalent interactions and promote the exposure of sulfhydryl groups, potentially through partial disruption of disulfide bonds [36], while high-shear forces can further prevent re-aggregation and stabilize the unfolded state [37]. This cooperative effect may increase the accessibility of protease cleavage sites, enhance hydrolysis efficiency, and favor the release of low-molecular-weight peptides, some of which may contain hydrophobic residues or sequences associated with bioactivity. These peptides may interact with α-glucosidase through hydrophobic interactions, hydrogen bonding, and other non-covalent forces, which may contribute to the observed inhibitory activity [35]. Therefore, particle size, surface hydrophobicity, and free sulfhydryl content may serve as useful structural indicators for describing the potential relationships among treatment-induced conformational changes, peptide release, and α-glucosidase-inhibitory activity.

These findings are consistent with previous reports. Liu et al. [38] demonstrated that ultrasound treatment was associated with the exposure of sulfhydryl groups and possible disruption of disulfide-related structures, along with increases in -SH content and surface hydrophobicity and a reduction in particle size, with free sulfhydryl content serving as an indicator of protein dispersion and structural accessibility. Huo et al. [20] further reported that ultrasound-assisted enzymolysis promotes protein unfolding and can favor the generation of short peptides containing hydrophobic residues, which may enhance bioactivity through multiple intermolecular interactions. Overall, these findings suggest a potential structure–function relationship in which structural disassembly and conformational loosening may enhance enzymatic digestibility and ultimately improve the in vitro α-glucosidase inhibitory activity of the resulting hydrolysates.

### 3.12 Decoding peptide-enzyme interactions through computational molecular docking

Digital 4.0 technologies are increasingly being integrated into food science to support data-driven ingredient discovery and accelerate the identification of bioactive compounds [39]. Molecular docking is one such computational approach that can help translate peptide sequence information into predicted three-dimensional interaction patterns and provide preliminary insights into peptide-enzyme recognition [40]. In this study, molecular docking was employed to characterize the potential interactions between peptides identified from *J. sigillata* protein hydrolysates and α-glucosidase, thereby supporting the selection of peptides for subsequent experimental validation. Traditional approaches for identifying inhibitory peptides from complex protein hydrolysates are labor-intensive and often lack mechanistic resolution. To improve the efficiency of candidate prioritization, LC-MS/MS-based sequence identification was integrated with a two-stage bioinformatics screening strategy and secondary verification using the BIOPEP-UWM database [[21], [41]]. This workflow was designed to reduce the need for indiscriminate screening of all peptides present in the hydrolysate and to prioritize candidates for experimental testing.

Based on LC-MS/MS sequence identification and two-tier bioinformatics-driven screening integrated with BIOPEP-UWM cross-verification, 20 high-potential candidates were prioritized by evaluating bioactivity probability, toxicity profiles, and allergenic risk (Table S4). To obtain preliminary structural insights into their potential inhibitory interactions, the eight top-ranked peptides, each with a PeptideRanker score > 0.80 and with no toxicity or allergenicity predicted by the applied *in silico* tools, were selected for molecular docking with α-glucosidase to evaluate their predicted structural complementarity and docking scores.

Under the applied docking conditions, more negative scores generally suggested more favorable predicted peptide-enzyme interactions and were used primarily as comparative computational estimates of interaction favorability. In some studies, docking scores below −4.0 kcal/mol have been used as a rough reference for favorable predicted interactions [42]; however, this threshold should be interpreted cautiously because docking scores are method-dependent estimates rather than experimental binding energies. As detailed in Table S5 and visualized in Fig. 5, six of the eight sequences showed comparatively favorable docking scores and plausible interaction patterns. Among them, the hexapeptide FFPGSP, the heptapeptide FGPSQPF, and the nonapeptide APSKDAPMF were selected for subsequent in vitro validation because they represented different peptide lengths and displayed relatively favorable docking scores and plausible interaction patterns. Three-dimensional (3D) docking poses suggested that these peptides could occupy regions near the reported catalytic pocket of α-glucosidase and form various hydrogen-bonding, hydrophobic, and other noncovalent interactions. Such interactions may interfere with substrate access or alter the local environment of the catalytic region, potentially contributing to α-glucosidase inhibition.

**Fig. 5:**
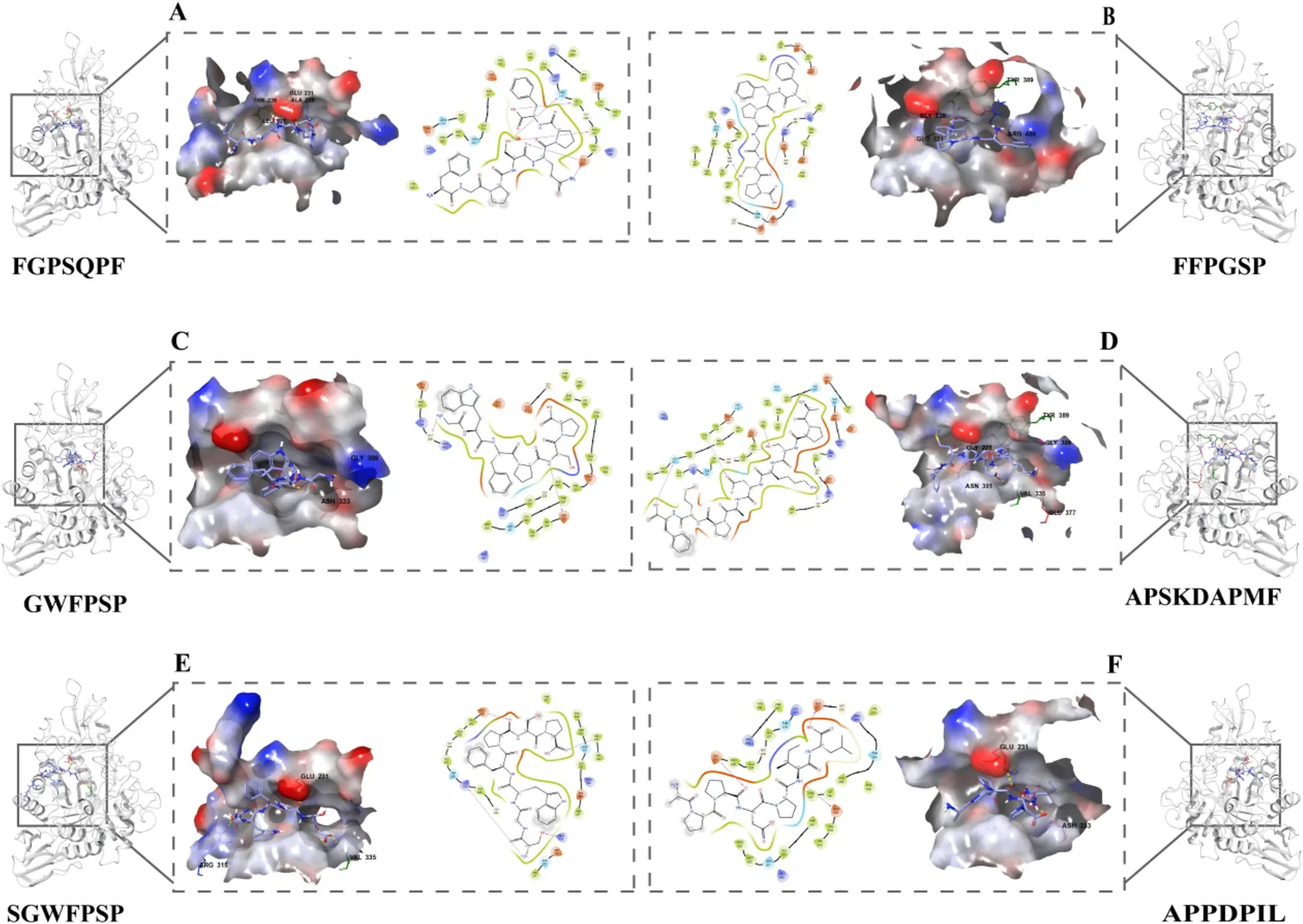
Molecular docking simulations of identified *J. sigillata* peptides with α-glucosidase. (A-F) Optimal binding conformations and 2D interaction diagrams for peptides (A) FGPSQPF, (B) FFPGSP, (C) GWFPSP, (D) APSKDAPMF, (E) SGWFPSP, and (F) APPDPIL. The central panels highlight the active site residues and specific bonding types (e.g., hydrogen bonds, hydrophobic interactions).

By contrast, sequences like APPDPILG and GGDPRPPGQPM showed less favorable predicted interactions, which may be related to unfavorable steric arrangements, limited contact formation, or greater conformational flexibility. Conversely, the peptides with more favorable docking scores were relatively enriched in Pro and Phe residues, which may have contributed to their predicted interaction profiles. The hydrophobic and aromatic properties of Phe may support nonpolar contacts, whereas the conformational constraints imposed by Pro may reduce backbone flexibility and facilitate peptide accommodation within the predicted binding region [43]. However, these residue-level interpretations remain hypotheses derived from static docking models and require experimental verification.

The present workflow illustrates how computational tools can be incorporated into a Digital 4.0-oriented strategy for bioactive peptide discovery. Specifically, LC-MS/MS provided peptide sequence information, bioinformatics tools supported the preliminary assessment of bioactivity and safety, and molecular docking supplied predicted structural descriptors, including docking scores, hydrogen-bonding patterns, and hydrophobic contacts. This integration converted complex sequence data into a ranked set of experimentally testable candidates, reducing the need for indiscriminate screening of all peptides present in the hydrolysate. Similar applications of digital and computational approaches have been reported for the discovery and mechanistic analysis of food-derived bioactive compounds [[39], [44]].

Molecular docking, however, provides a simplified view of peptide–enzyme interactions and may not fully capture receptor flexibility, solvent effects, protonation states, entropy, or the dynamic nature of binding. In this study, the docking results were therefore used to guide candidate selection and suggest possible interaction patterns. The subsequent in vitro dose–response experiments helped determine whether these selected peptides exhibited α-glucosidase inhibitory activity.

### 3.13 Molecular dynamics simulation analysis

MD simulations were performed to examine the temporal stability and conformational evolution of the peptide-enzyme complexes [45]. MD trajectories confirmed that FFPGSP remained stably bound to α-glucosidase, maintaining a coherent and integrated complex structure throughout the 100 ns simulation [46]. The root-mean-square deviation (RMSD) profiles (Fig. 6A) demonstrated that the FFPGSP-enzyme system attained conformational equilibrium rapidly within 30 ns [47]. The subsequent minor fluctuations in both the protein backbone and the ligand indicate high structural integrity. Localized mobility analysis via root-mean-square fluctuation (RMSF) showed that residues in the interaction interface exhibited markedly suppressed fluctuations (Fig. 6B), confirming that the peptide effectively “locked” the active-site conformation [48]. The stability of the radius of gyration (*Rg*) and solvent-accessible surface area (SASA) (Figs. 6C and 6D) further verified the maintenance of a compact global architecture, with SASA stabilizing around 21,000–21,500 Å^2^. Non-covalent interactions were dominated by a persistent network of hydrogen bonds and π-π stacking (Fig. 6E), driven by a hydrophobicity-dominated binding motif [49]. The contact frequency remained high (4–16) without any dissociation trends (Fig. 6F), with ASP-333, GLU-271, ARG-400, and PHE-397 identified as the primary anchoring residues.

**Fig. 6:**
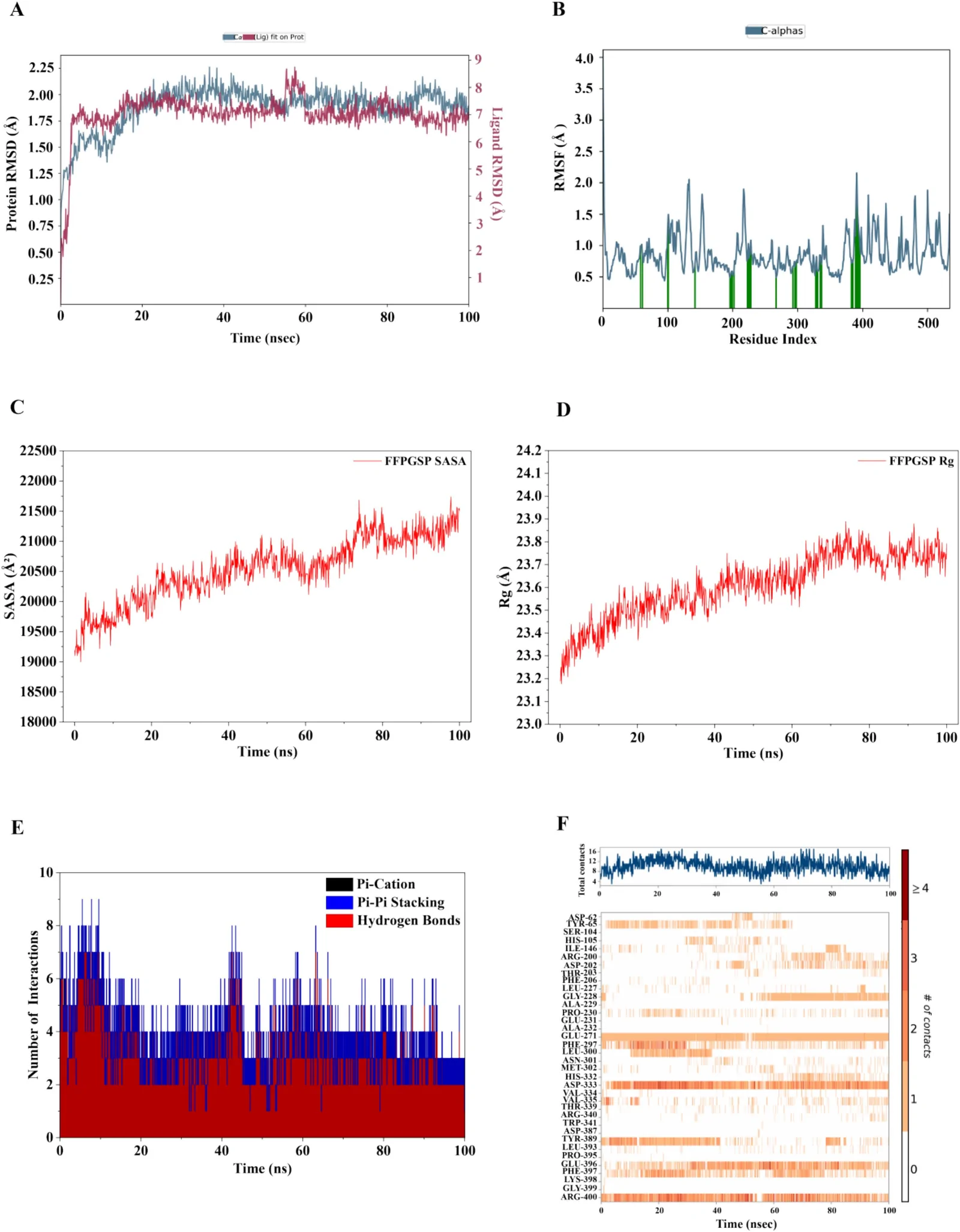
Molecular dynamics (MD) simulation analysis of the FFPGSP-α-glucosidase complex. (A) RMSD profiles for the protein backbone and the ligand over a 100 ns trajectory; (B) RMSF of protein residues; (C) Solvent accessible surface area (SASA); (D) Radius of gyration (*Rg*); (E) Evolution of intermolecular hydrogen bonds, π-π stacking, and π-cation interactions; (F) Heatmap of contact frequencies between the peptide and enzyme residues.

For the APSKDAPMF complex, equilibrium was reached at approximately 40 ns (Fig. 7A). The system exhibited steady dynamic behavior without abrupt structural deformations, and residue flexibility at the interface was significantly restrained (Fig. 7B). The primary driving forces included hydrogen bonding, supplemented by π-cation and salt-bridge interactions (Fig. 7E). A multistage anchoring process was observed (Fig. 7F), initiating at LYS-225 and maturing into a stable secondary binding state via HIS-304 and ASP-344.

**Fig. 7:**
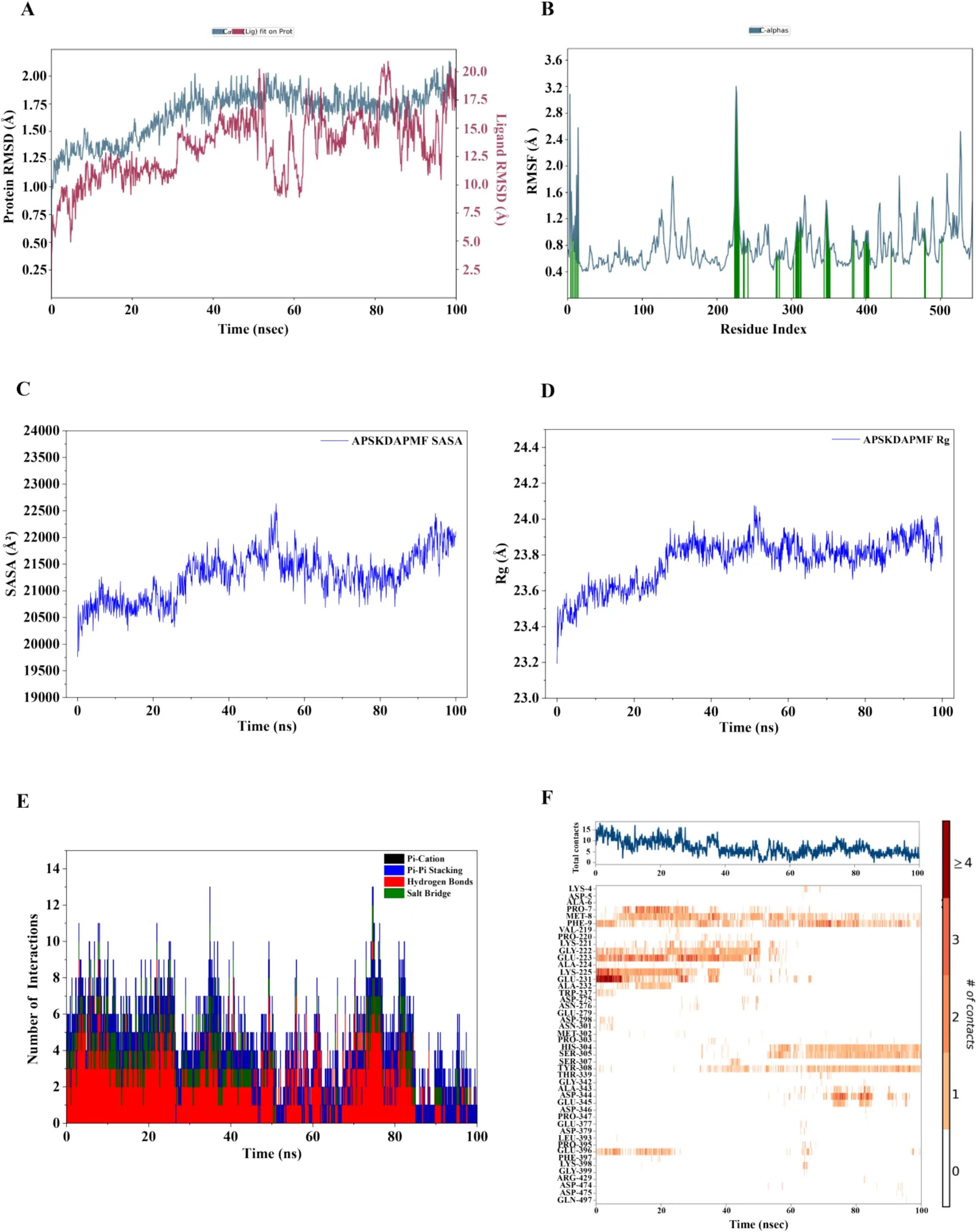
Molecular dynamics (MD) simulation analysis of the APSKDAPMF-α-glucosidase complex. (A) RMSD of protein and ligand; (B) RMSF of residue fluctuations; (C) SASA; (D) *Rg* evolution; (E) Number of dynamic interactions (hydrogen bonds, salt bridges, etc.); (F) Residue-level contact heatmap illustrating binding stability.

**Fig. 8:**
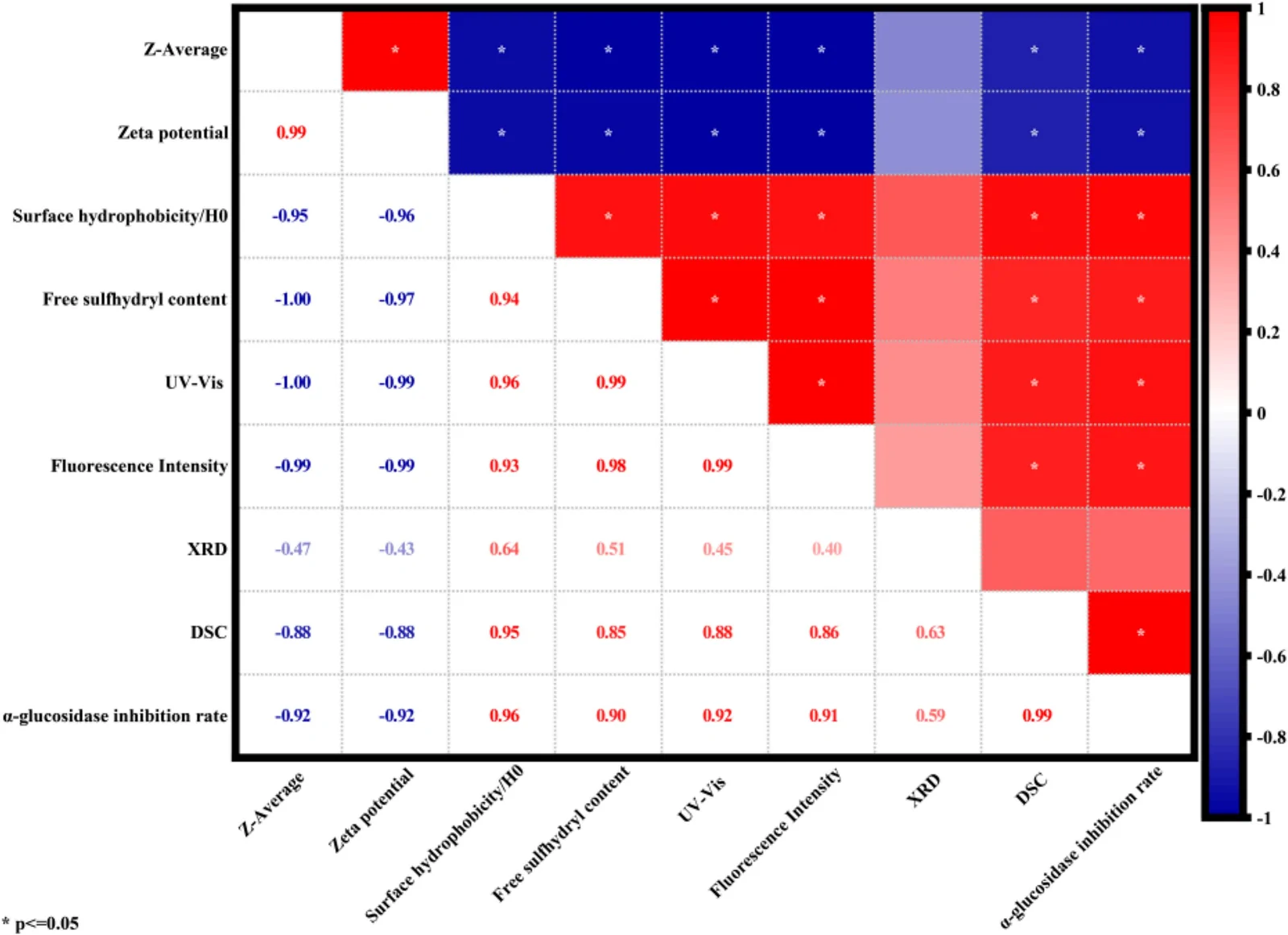
A heatmap showing the correlation coefficients between physicochemical/structural properties and α-glucosidase inhibitory activity of *J. sigillata* protein and its hydrolysates. Cells display Pearson’s r; * *p* < 0.05.

The contrasting dynamic behaviors between FFPGSP (hexapeptide) and APSKDAPMF (nonapeptide) highlight the influence of peptide chain length on binding stability. Shorter peptides, such as FFPGSP, possess fewer conformational degrees of freedom, which restricts backbone flexibility and favors a more defined, rigid binding mode within the α-glucosidase active pocket [43]. This entropic advantage enables faster stabilization, as evidenced by RMSD equilibration within 30 ns, and reduces the likelihood of unfavorable intra-peptide interactions that could disrupt binding. In contrast, longer peptides like APSKDAPMF exhibit greater conformational plasticity, allowing them to adapt to the active site through multiple anchoring residues (e.g., LYS-225, HIS-304, ASP-344). However, this increased flexibility also leads to a longer equilibration time (∼40 ns) and a more dynamic interaction network, as reflected by the diverse polar contacts (hydrogen bonds, π-cation, salt bridges) observed during the simulation [45]. Thus, while both lengths can form stable complexes, shorter peptides tend to confer higher dynamic rigidity and faster binding kinetics, which may be advantageous for sustained enzyme inhibition. Together, these MD results provide dynamic evidence for the docking-predicted binding modes and extend the computational workflow from static structure prediction to dynamic stability assessment.

### 3.14 *In vitro* validation of synthetic peptides

To experimentally validate the α-glucosidase inhibitory activity of the candidate peptides identified by *in silico* screening, the three selected candidate peptides (FFPGSP, FGPSQPF, and APSKDAPMF) were chemically synthesized (Fig. S2). All three peptides exhibited concentration-dependent α-glucosidase inhibition, with IC_50_ values of 1745, 5393, and 4090 μM for FFPGSP, FGPSQPF, and APSKDAPMF, respectively. Because a lower IC_50_ value indicates stronger inhibitory potency, the peptides showed the following potency order: FFPGSP > APSKDAPMF > FGPSQPF. Although the experimental results generally supported the computational prediction that these peptides possess inhibitory potential, the experimentally determined potency order did not fully correspond to the ranking predicted by docking.

Notably, although APSKDAPMF exhibited the most favorable docking score, its IC_50_ value was higher than that of FFPGSP. This discrepancy may be related to steric effects associated with the longer peptide chain, differences between the solution-phase and docked conformations, or limitations of the static docking model. From a structural perspective, the relatively strong inhibitory activity of FFPGSP may be associated with its two N-terminal phenylalanine residues. Previous studies have suggested that N-terminal amino acid residues, particularly aromatic or hydrophobic residues, may play important roles in the α-glucosidase inhibitory activity of peptides [44].

The IC_50_ values obtained for the three peptides were lower than the reported IC_50_ value of VVDW from walnut (11.17 mM) [50]. In particular, the IC_50_ of FFPGSP was approximately 6.4-fold lower than that of VVDW. FFPGSP also showed a lower reported IC_50_ value than LDNFR from wheat germ (9.21 mM) [51]. Nevertheless, these comparisons should be treated with caution because the enzyme sources and assay conditions differed among studies. The dose–response data and IC_50_ values nevertheless supported the effectiveness of the screening strategy in identifying α-glucosidase-inhibitory peptides.

### 3.15 Comparative Analysis and Process Innovations

Integrating the structural and physicochemical results, the ultrasound-high-shear-assisted enzymatic hydrolysis strategy effectively remodeled the *J. sigillata* protein matrix. Compared with the untreated control, the S + U + E group exhibited the smallest particle size and the most negative zeta potential, indicating enhanced dispersion stability, reduced aggregation, and stronger electrostatic repulsion [[52], [53]]. Such changes were likely driven by the synergistic effects of ultrasound cavitation and high-shear processing. Ultrasonic cavitation generates localized high-energy microjets and shock waves that can disrupt weak intermolecular interactions and fragment compact protein aggregates [54], while rotor-stator-induced shear and turbulence further intensify mechanical disruption of the protein matrix [15]. In addition, the enlarged interfacial area resulting from these physical forces may improve enzyme accessibility and facilitate subsequent hydrolytic cleavage [20].

In parallel, the increased surface hydrophobicity and free sulfhydryl content further suggested protein unfolding. The rise in hydrophobicity likely resulted from cavitation-induced exposure of buried hydrophobic residues and the generation of smaller peptide fragments during enzymatic hydrolysis [[38], [55]]. Meanwhile, the elevated free sulfhydryl content implies that the combined physical pretreatments disrupted intramolecular interactions and exposed previously buried thiol groups, leading to a more extended protein conformation. The enzymatic action may further facilitate conformational loosening by cleaving susceptible peptide bonds and exposing reactive side chains [30].

These structural alterations were further supported by spectroscopic evidence. FTIR analysis showed weakened and broadened amide I bands, indicating partial disruption of ordered secondary structures and chain rearrangement [[56], [57]]. In addition, UV-Vis and fluorescence spectra revealed greater exposure of aromatic residues, reflecting perturbation of the hydrophobic core and increased molecular flexibility [58].

XRD and DSC further confirmed that the composite treatment altered protein ordering. The reduced diffraction intensity and broadened peaks indicated decreased structural order and a loss of long-range order, consistent with a transition toward a more amorphous state [59]. The more concentrated endothermic peak observed in the S + U + E group suggests a more homogeneous thermal response, likely resulting from the synergistic effects of shear, ultrasound, and enzymatic cleavage on protein reorganization [[60], [61], [62]]. Taken together, these results indicate that the ultrasound-high-shear-assisted enzymatic hydrolysis strategy effectively remodeled the *J. sigillata* protein matrix. Compared with the single or dual physical pretreatment strategies previously applied to soybean and pea proteins [41], the present system provided a more pronounced disruption of the compact protein matrix, thereby facilitating unfolding, reducing aggregation, and exposing reactive sites for subsequent peptide release.

## 4 Conclusion

This study demonstrated that an ultrasound-shear-assisted enzymolysis strategy effectively promoted the release of α-glucosidase-inhibitory peptides from *J. sigillata* protein. Under RSM-optimized conditions of 21 min ultrasound treatment at 400 W, 12000 r/min shearing speed and enzyme dosage of 12 KU/g, the hydrolysate exhibited significantly higher α-glucosidase inhibitory activity than conventional single enzymolysis. Multiscale structural analyses, including SEM, XRD, FTIR, DSC and spectroscopic characterization, collectively indicated that the coupled ultrasound–high-shear treatment induced partial disruption and rearrangement of ordered protein structures, reduced particle size, and increased the exposure of hydrophobic and free sulfhydryl groups. These changes likely enhanced protease accessibility to susceptible peptide bonds and improved the overall hydrolysis performance. LC–MS/MS identified 4,492 peptide sequences, and an integrated screening strategy based on bioinformatics analysis was used to select candidate α-glucosidase-inhibitory peptides. Molecular docking and molecular dynamics simulations were conducted for FFPGSP and APSKDAPMF to further investigate their binding behavior with α-glucosidase. The simulation results indicated that FFPGSP displayed a more favorable interaction profile than APSKDAPMF, as reflected by its more stable binding conformation and stronger non-covalent interactions with key residues of α-glucosidase. These findings were consistent with the in vitro inhibition assay, in which FFPGSP showed the highest α-glucosidase inhibitory activity, with an IC_50_ value of 1745 μM, while APSKDAPMF and FGPSQPF exhibited IC_50_ values of 4090 and 5393 μM, respectively.

Overall, the ultrasound–high-shear-assisted enzymatic hydrolysis process provides a promising approach for releasing α-glucosidase-inhibitory peptides from *J. sigillata* protein. The agreement between computational screening and experimental validation supports the feasibility of the proposed peptide-discovery workflow. Further research should assess the gastrointestinal stability, bioavailability, and *in vivo* efficacy of these peptides, while the integration of AI-assisted prediction and real-time process monitoring may facilitate process optimization and industrial scale-up.

## CRediT authorship contribution statement

**Xingjing Zhang:** Writing – original draft. **Boxiao Wu:** Resources. **Baitong Zhu:** Methodology. **Zhongxing Chu:** Supervision. **Quankang Liu:** Data curation. **Chang Zou:** Resources. **Ting Ma:** Project administration. **Feijun Luo:** Writing – review & editing. **Yaping Zhou:** Writing – review & editing. **Delu Ning:** Writing – review & editing, Funding acquisition.

## Declaration of competing interest

The authors declare that they have no known competing financial interests or personal relationships that could have appeared to influence the work reported in this paper.

## Appendix A Supplementary data

Supplementary Data 1 The supplementary material includes the Box–Behnken design parameters, ANOVA results, peptide activity and safety scores, molecular docking scores, and α-glucosidase inhibitory activity data for the three synthetic peptides.
