# Supplementary material for: Ultrasound-shear-assisted enzymatic preparation of α-glucosidase inhibitory peptides from Juglans sigillata D. and elucidation of their molecular inhibitory mechanism
Source: Ultrason Sonochem. 2026 Sep 2;133:108034. doi: 10.1016/j.ultsonch.2026.108034 (PMC13673472; doi:10.1016/j.ultsonch.2026.108034)
Supplement: Supplementary file 1 [file mmc1.docx]

**Supplementary data**

Table S1 Factors and levels of Box-Behnken experiment design

| Factor | Levels | | |
| --- | --- | --- | --- |
|  | -1 | 0 | 1 |
| A: Time (min) | 10 | 20 | 30 |
| B: Ultrasonic power (W) | 300 | 400 | 500 |
| C: Enzyme addition amount (KU/g) | 10 | 14 | 18 |

Table S2 The Box-Behnken experimental design and results

| Number | Enzyme addition amount (KU/g) | Ultrasonic power (W) | Time (min) | α-Glucosidase inhibition rate (%) |
| --- | --- | --- | --- | --- |
| 1 | -1 | 0 | 1 | 45.93 |
| 2 | 0 | -1 | -1 | 46.1 |
| 3 | 0 | 1 | 1 | 48.32 |
| 4 | 1 | 0 | 1 | 48.96 |
| 5 | 1 | 0 | -1 | 46.43 |
| 6 | -1 | -1 | 0 | 45.48 |
| 7 | -1 | 0 | -1 | 45.15 |
| 8 | 1 | -1 | 0 | 47.53 |
| 9 | 0 | 0 | 0 | 57.55 |
| 10 | 0 | -1 | 1 | 49.45 |
| 11 | 1 | 1 | 0 | 48.21 |
| 12 | 0 | 0 | 0 | 57.88 |
| 13 | 0 | 0 | 0 | 58.25 |
| 14 | 0 | 1 | -1 | 48.75 |
| 15 | -1 | 1 | 0 | 45.98 |

Table S3 Analysis of variance and significance test of regression model

| Source | Sum of Squares | DF | Mean Square | F-value | p-value | Signific-ant |
| --- | --- | --- | --- | --- | --- | --- |
| Model | 299.88093167 | 9 | 33.32010352 | 597.47353968 | 4.8878301209e-07 | *** |
| A-Enzyme addition amount | 9.2235125 | 1 | 9.22351250 | 165.38978213 | 5.0617589703e-05 | *** |
| B-Ultrasonic power | 0.91125000 | 1 | 0.91125000 | 16.33991811 | 0.0099005678 | ** |
| C-Time | 4.85161250 | 1 | 4.85161250 | 86.99583097 | 0.0002385091 | *** |
| AB | 0.00810000 | 1 | 0.00810000 | 0.14524372 | 0.7187791545 |  |
| AC | 0.76562500 | 1 | 0.76562500 | 13.72866920 | 0.0139237364 | * |
| BC | 3.57210000 | 1 | 3.57210000 | 64.05247901 | 0.0004919604 | *** |
| A² | 147.26580064 | 1 | 147.26580064 | 2640.67064298 | 5.2754288567e-08 | *** |
| B² | 84.28980064 | 1 | 84.28980064 | 1511.42764366 | 2.1220798909e-07 | *** |
| C² | 90.85193910 | 1 | 90.85193910 | 1629.09546820 | 1.7602950872e-07 | *** |
| Residual | 0.27884167 | 5 | 0.05576833 |  |  |  |
| Lack of Fit | 0.03357500 | 3 | 0.01119167 | 0.09126121 | 0.9582181633 |  |
| Pure Error | 0.24526667 | 2 | 0.12263333 |  |  |  |
| Cor Total | 300.15977333 | 14 |  |  |  |  |
| R^2^ | 0.9991 |  |  |  |  |  |
| R^2^Adj | 0.9974 |  |  |  |  |  |
| R^2^Pred | 0.9964 |  |  |  |  |  |
| C.V.% | 0.4787 |  |  |  |  |  |
| S/N | 65.82 |  |  |  |  |  |

Table S4 Results of score data of different walnut peptides

| No. | Peptide | Activity score | Hydrophobicity score | Toxicity | Sensitizing potential | Free radical scavenger (FRS) | Chelation score |
| --- | --- | --- | --- | --- | --- | --- | --- |
| 1 | GWFPSP | 0.965022 | -0.575 | Non-Toxin | Non-Allergen | 0.50831056 | 0.26111582 |
| 2 | SGWFPSP | 0.952263 | -2.098 | Non-Toxin | Non-Allergen | 0.50409549 | 0.23654953 |
| 3 | FFPGSP | 0.938449 | -1.056 | Non-Toxin | Non-Allergen | 0.47895589 | 0.29624745 |
| 4 | FGPSQPF | 0.923312 | -2.306 | Non-Toxin | Non-Allergen | 0.45564944 | 0.28176677 |
| 5 | VPPFLP | 0.915864 | 1.286 | Non-Toxin | Probable Allergen | 0.46839041 | 0.27971882 |
| 6 | APPDPILG | 0.908507 | -1.471 | Non-Toxin | Non-Allergen | 0.4180755 | 0.24836558 |
| 7 | APPDPIL | 0.883154 | -0.587 | Non-Toxin | Non-Allergen | 0.43877962 | 0.27343839 |
| 8 | SFQNIF | 0.878313 | -2.513 | Non-Toxin | Probable Allergen | 0.36235768 | 0.24858552 |
| 9 | YVPPFLP | 0.874842 | 1.719 | Non-Toxin | Probable Allergen | 0.51954627 | 0.25111389 |
| 10 | GGDPRPPGQPM | 0.860292 | -6.078 | Non-Toxin | Non-Allergen | 0.54708958 | 0.29159534 |
| 11 | RFFPGSP | 0.852712 | -2.308 | Non-Toxin | Probable Allergen | 0.45295164 | 0.28336322 |
| 12 | APSKDAPMF | 0.851488 | -3.079 | Non-Toxin | Non-Allergen | 0.36579737 | 0.21302365 |
| 13 | SFPAPA | 0.850602 | -1.89 | Non-Toxin | Non-Allergen | 0.42560062 | 0.31156743 |
| 14 | VLFFGGP | 0.82365 | 0.264 | Non-Toxin | Non-Allergen | 0.46601471 | 0.24962084 |
| 15 | AFPSPSPDGK | 0.801806 | -5.238 | Non-Toxin | Non-Allergen | 0.44644687 | 0.25631845 |
| 16 | KVPGFPRP | 0.799658 | -1.767 | Non-Toxin | Probable Allergen | 0.46920109 | 0.24078311 |
| 17 | IVPPPLPTPPPG | 0.791895 | -0.965 | Non-Toxin | Non-Allergen | 0.5400247 | 0.27214849 |
| 18 | LPKPYLPR | 0.782153 | -0.257 | Non-Toxin | Probable Allergen | 0.50900066 | 0.22207372 |
| 19 | SLPKPYLPR | 0.770605 | -1.78 | Non-Toxin | Probable Allergen | 0.47648847 | 0.23187611 |
| 20 | GNQGFEW | 0.741677 | -3.458 | Non-Toxin | Non-Allergen | 0.43425608 | 0.20201105 |

Table S5 The binding ability between the peptide sequence and the Alpha-glucosidase receptor

| ID | Peptide | docking score |
| --- | --- | --- |
| 1 | GWFPSP | -7.517 |
| 2 | SGWFPSP | -7.425 |
| 3 | FFPGSP | -9.925 |
| 4 | FGPSQPF | -9.652 |
| 5 | APPDPILG | / |
| 6 | APPDPIL | -7.163 |
| 7 | GGDPRPPGQPM | / |
| 8 | APSKDAPMF | -10.185 |

Fig. S1. α-Glucosidase inhibitory activity of the three synthetic peptides (FFPGSP, FGPSQPF, and APSDKAPMF) at a single concentration of 0.5 mM. Data are expressed as mean ± SD (n = 3). Different lowercase letters indicate significant differences (*p* < 0.05).

Fig. S2 Dose-response curves of α-glucosidase inhibition by FFPGSP, APSKDAPMF, and FGPSQPF. Data are expressed as mean ± SD (n = 3). The R² values for the three fits were 0.9714 (FFPGSP), 0.9783 (APSKDAPMF), and 0.9616 (FGPSQPF).
